# A large upper abdominal mass in an adolescent with high Ca 19.9: a case report

**DOI:** 10.1093/jscr/rjac186

**Published:** 2022-04-27

**Authors:** Robert M O’Connell, Adrian O’Sullivan

**Affiliations:** Department of Hepatopancreaticobiliary Surgery, Mercy University Hospital, Cork, T12 WE28, Ireland; Department of Hepatopancreaticobiliary Surgery, Mercy University Hospital, Cork, T12 WE28, Ireland

## Abstract

Mucinous cystic neoplasms of the liver are uncommon cystic lesions of the liver, most commonly seen in women in the fifth decade of life. We present a case of a 16-year-old girl with an incidentally discovered abdominal mass while undergoing a tonsillectomy. Investigation revealed a multiloculated, septated 17 × 17 × 11 cm cystic lesion arising from the left lobe of the liver, with displacement of the remaining upper abdominal viscera. Serum Ca19.9 was significantly elevated at 2256 U/ml (range 0–37), but other bloods including liver function tests, alphafoetoprotein and carcinoembryonic antigen were within normal limits. We proceeded to open formal left hemi-hepatectomy. Histology was consistent with a diagnosis of mucinous cystic neoplasm with low-grade intra-epithelial neoplasia.

## INTRODUCTION

Mucinous cystic neoplasms of the liver (MCN-L), previously known as hepatobiliary cystadenoma or cystadenocarcinoma, are uncommon cystic tumours that account for up to 5% of hepatic cystic lesions, although the true incidence may be lower than that [1]. MCN-L are typically encountered in young women and more commonly found in the left lobe of the liver [2]. Distinguishing MCN-L from other cystic neoplasms of the liver such as simple cysts, cystic hamartomas and intraductal papillary neoplasms of the bile duct is important, as MCN-L have invasive potential, but remains challenging—particularly as only a limited number of cases have been reported in the literature [3]. The treatment of MCN-L is resection, particularly where invasive disease is suspected, or careful enucleation as recurrence rates as high as 59% are seen with incomplete excision [4].

## CASE DISCUSSION

This 16-year-old lady was referred to our service following an incidental finding of an upper abdominal mass, noted by an anaesthetist following administration of a general anaesthetic for an elective tonsillectomy for recurrent tonsillitis. She was a smoker and taking a combined oral contraceptive pill for the treatment of acne vulgaris, but her medical history was otherwise unremarkable.

On initial outpatient evaluation, she denied any symptoms associated with the mass, described herself as well and denied any weight loss. Physical examination revealed a palpable, non-tender mass in the epigastrium, measuring approximately 15 × 15 cm. Initial bloods taken showed a normal full blood count, liver function tests and urea and electrolytes. Ca19.9 was elevated at 2256 U/ml (range 0–37), but alphafoetoprotein (AFP, <2.0 ng/ml, range 0–8.8) and carcinoembryonic antigen (1.0 ng/ml, range 0–5.0) were within normal limits. Serum amoebic antibodies and echinococcus antibodies were negative.

**Figure 1:**
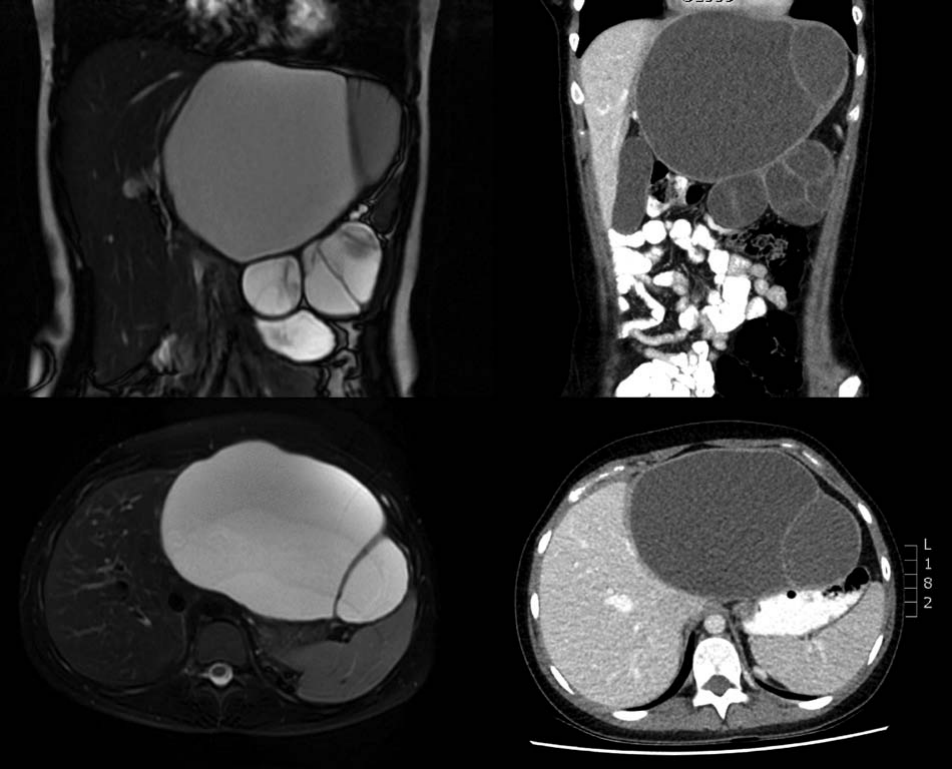
MRI (left) and CT (right) preoperative imaging.

Imaging was undertaken with ultrasound, magnetic resonance imaging (MRI) of liver, and computed tomography (CT) of thorax, abdomen and pelvis. A loculated 17 × 17 × 11 cm cystic lesion was noted in the upper abdomen, arising from the left lobe of the liver with negligible amount of parenchyma remaining in the left lateral section with displacement of the pancreas, stomach, hepatic flexure and spleen (Fig. 1). No lymphadenopathy or metastatic lesion was seen.

**Figure 2:**
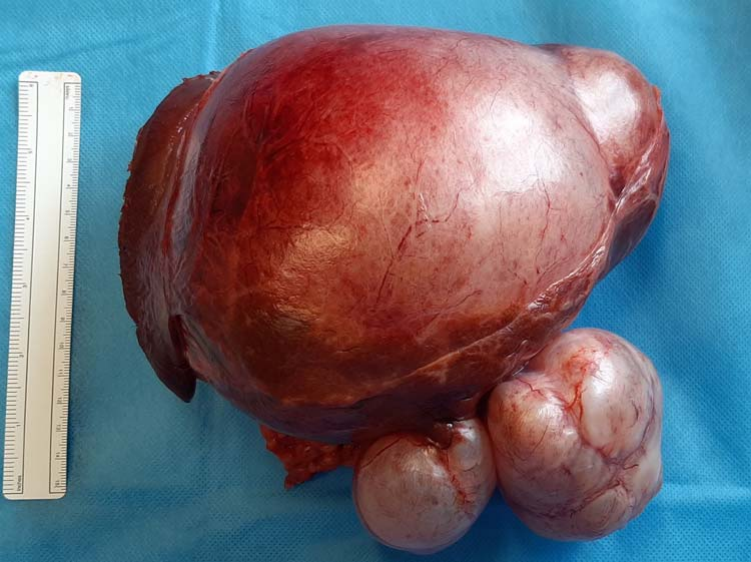
Resected left hepatectomy specimen showing the large, multiloculated cystic lesion arising from the left lobe of the liver.

Following discussion at our interdepartmental multidisciplinary meeting, we proceeded with a formal left hemihepatectomy through a subcostal incision. Intermittent pringle was utilized for haemostasis, and the transection plane was through Cantlie’s line. At time of surgery, a large dominant cyst was noted, occupying the left lobe of her liver, with complex loculations extending inferiorly but contiguous with the liver and not invading adjacent structures, weighing 1807 g (Fig. 2).

Her postoperative course was uncomplicated and was discharged home on postoperative day 6.

Histology showed a multilocular cyst with cuboidal to columnar lining and intraluminal mucin. This was consistent with a mucinous cystic neoplasm with low-grade intra-epithelial neoplasia. There was complete excision of the cyst (R0).

She was well on outpatient review 6 weeks later, her wounds had healed well and her serum Ca19.9 levels had returned to a normal level (14.0 U/ml, range 0–37). She was discharged from follow-up.

## DISCUSSION

This case represents many features consistent with other cases of MCN-L reported in the literature; however, there are some unique elements. Not least among these is the patient’s age at presentation was 16 years, whereas MCN-L are most commonly seen in the fifth decade of life and rarely under 21 years [2]. While the majority of patients with MCN-L will present symptomatically, a significant minority, such as our patient, will have incidentally discovered lesions [5].

Of some concern at presentation, this patient had an elevated serum Ca 19-9 level. This has been described in non-invasive MCN-L previously, particularly in the presence of obstructive jaundice, but may be associated with invasive disease [5, 6]. As reported above, her serum Ca 19-9 level returned to within normal range at clinical follow-up.

Imaging remains the cornerstone of preoperative diagnosis of MCN-L. Ultrasound is often the first modality of choice—it enables the differentiation between simple hepatic cysts and more structurally complex cystic neoplasms, but it is difficult to differentiate between invasive and non-invasive MCN-L [7]. Classical findings on CT and MRI were seen in this case: a large, solitary, multiloculated and septated cystic lesion, with a well-circumscribed margin [8]. Differentiation between invasive and non-invasive on cross-sectional imaging is also problematic. Features such as mural nodularities, intracyst haemorrhage and calcification of the cyst wall or septations may suggest an invasive neoplasm [8].

As in this case, MCN-L are definitively diagnosed based on histological findings. The World Health Organization classification defines MCN-L as cyst-forming epithelial neoplasms comprising cuboidal to columnar, variably mucin-producing epithelium, with the presence of ovarian-type subepithelial stroma [9]. MCN-L are categorized based on the highest degree of atypia into MCN-L with low-grade intraepithelial neoplasia (as here), high-grade intraepithelial neoplasia or with an associated invasive carcinoma.

The overall prognosis for MCN-L in the absence of invasive disease is excellent. Arnaoutakis *et al*. [4] report an overall recurrence rate of 9.5% for non-invasive MCN-L undergoing R0 resection. Significant long-term survival of 97% at 11 years has been reported in the literature [5].

## CONFLICT OF INTEREST STATEMENT

None declared.

## FUNDING

This research did not receive any specific grant from funding agencies in the public, commercial, or not-for-profit sectors.

## STATEMENT OF AUTHORSHIP

All named authors made substantial contributions to this work, sufficient to meet the guidelines of the International Committee of Medical Journal Editors (ICMJE) of appropriate authorship.
